# Anti-Mullerian Hormone:* Above and Beyond Conventional Ovarian Reserve Markers*

**DOI:** 10.1155/2016/5246217

**Published:** 2016-02-10

**Authors:** Zehra Jamil, Syeda Sadia Fatima, Khalid Ahmed, Rabia Malik

**Affiliations:** Department of Biological & Biomedical Sciences, Aga Khan University, P.O. Box 74800, Karachi, Pakistan

## Abstract

Management of ovarian dysfunctions requires accurate estimation of ovarian reserve (OR). Therefore, reproductive hormones and antral follicle count (AFC) are assessed to indicate OR. Serum anti-Mullerian hormone (AMH) is a unique biomarker that has a critical role in folliculogenesis as well as steroidogenesis within ovaries. Secretion from preantral and early antral follicles renders AMH as the earliest marker to show OR decline. In this review we discuss the dynamics of circulating AMH that remarkably vary with sex and age. As it emerges as a marker of gonadal development and reproductive disorders, here we summarize the role of AMH in female reproductive physiology and provide evidence of higher accuracy in predicting ovarian response to stimulation. Further, we attempt to compile potential clinical applications in children and adults. We propose that AMH evaluation has a potential role in effectively monitoring chemotherapy and pelvic radiation induced ovarian toxicity. Furthermore, AMH guided ovarian stimulation can lead to individualization of therapeutic strategies for infertility treatment. However future research on AMH levels within follicular fluid may pave the way to establish it as a marker of “quality” besides “quantity” of the growing follicles.

## 1. Introduction

Sexually undifferentiated embryo consists of Wolfian as well as Mullerian ducts. In males, the testicular Sertoli cells start secreting anti-Mullerian hormone (AMH) as early as 7th week of gestation that leads to regression of Mullerian duct. As a result, the Wolfian duct gives rise to epididymis and the seminal vesicles, under the influence of testosterone. Since then, AMH remains steady until puberty, when it rapidly declines in response to testosterone synthesis [1]. On the other hand, absence of AMH in female fetus allows the Mullerian duct to give rise to uterus, fallopian tubes, and upper part of vagina. The earliest production of AMH in females is reported at the 36th week of gestation. At birth, they have approximately 35 times lower AMH than males of similar age [1]. Since then, gradual surge in the production of AMH reflects a steady rise in the number of growing ovarian follicles. The strong correlation of AMH with the number of growing follicles is supported by the fact that its levels are reported very high in ovarian tumors [2] and in polycystic ovaries [3] while undetectable levels are testified in postmenopausal women [4] and Turner syndrome patients without gonadal tissue [1]. Regarding the production of AMH in later life, a mild peak is observed at the puberty, followed by the highest level of secretion between 23 and 25 years of age [5]. This corresponds to the most fertile era of a female. Afterwards, these levels steadily decline until the hormone becomes undetectable, corresponding to menopause [5]. Over recent years, researchers have highlighted AMH as a valid marker of ovarian ageing [6].

AMH, a homodimeric glycoprotein, is also recognized as Mullerian inhibiting hormone (MIH). It belongs to the family of transforming growth factor-*β* (TGF-*β*) and its gene is located on chromosome 19 p13.3, containing 5 exons [1]. The hormone binds to its receptor (AMHR), a single transmembrane protein with serine-threonine kinase activity [7]. These receptors are expressed on target organs such as Mullerian ducts, Sertoli and Leydig cells of testis, and granulosa cells of the ovary. Several genes have been identified that regulate the production of AMH such as* SF1*,* GATA1*,* WT1, DAX1*, and* SOX9* [8]. AMH has been conventionally known for its role in male sexual differentiation until the late 1990s, when it was identified and reported for the first time in females [9].

## 2. AMH in Reproductive Physiology

### 2.1. Ovarian Physiology

Females are born with a fixed number of primordial follicles, resting in a dormant state of meiosis II until puberty, until they enter different stages of development. The quantity and the quality of primordial follicle constitute the reserve of an ovary [10]. Due to the size and placement of these resting follicles, direct assessment of the pool is nonfeasible. Although these dormant primordial follicles do not secrete AMH; however, as soon as they are recruited for development, expression for AMH secretion is reported [11]. Immunohistochemistry reveals that preantral and small antral follicles measuring 2 to 8 mm express highest amount of AMH thus making it the earliest marker of ovarian follicular growth [12]. As soon as these follicles enter FSH-dependent stages of development (8 to 10 mm size), this expression is said to be lost [13].

**Figure 1:**
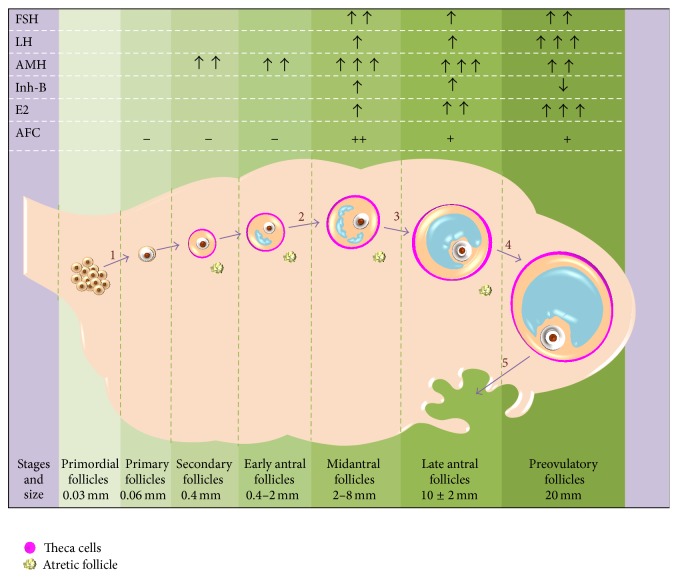
Schematic representation of hormonal surges across the follicular phase of ovarian cycle. 1: initial recruitment; 2: cyclic recruitment; 3: selection; 4: dominance; 5: ovulation. (−) non/poorly visualized; (+) well visualized. AMH is secreted by all growing follicles but its serum levels reflect only the secretion from follicles lying close to the vascular bed. It has an inhibitory effect on steps 1 and 2, thus maintaining the ovarian pool. The emphasis is on AMH production in early stages of follicle development as opposed to all other hormones that are released at later stages. Follicular fluid AMH levels show a better correlation with oocyte competence and hence can prove to be a reliable marker of embryo transfer outcomes.

AMH has a potential role in conservation of OR; it does so by exerting dual actions. Firstly, it inhibits the initial recruitment of follicles for growth by averting several stimulatory growth factors for recruitment such as KIT ligand and basic fibroblast growth factor [14]. Secondly, since puberty, AMH reduces the sensitivity of the primordial follicles to FSH, thus decreasing their chance of cyclic recruitment [15]. Once a follicle reaches the size of around 8 mm and is selected for dominance, AMH production rapidly declines [12]. This supports the role of AMH as a major regulator of initial as well as cyclic recruitment of follicles by maintaining their threshold for FSH sensitivity (Figure 1). This is further evident by the fact that, in AMH null mice, the greater number of types of follicular recruitment leads to a burnout of primordial pool at an earlier age [15].

### 2.2. Physiology of Menstrual Cycle

During menstrual cycle it is observed that AMH exhibit mild fluctuation; however this intercyclic variation is much lower than its variability amongst individuals of the same age (also termed as interindividual variability) [16]. Although in recent times, limited studies have reported intercyclic fluctuation as these variations are observed at random times, majority of the researchers provide convincing evidence that AMH can be measured at any time throughout the cycle [17]. Mild fluctuation can be explained by the fact that dominant follicles lack AMH production, resulting in slight decline during late follicular stage. Furthermore, interindividual variability mainly reflects the greater difference of OR amongst individuals. Few studies have also reported ethnic variations, suggesting discrepancy between OR amongst different populations [18]. This fact calls for a need to establish valid baselines of AMH levels in diverse population across the world. Currently, in women aged 25 to 40 years, literature in terms of fertility recommends levels of 1.0 to 3.0 ng/mL AMH as “normal,” 0.7 to 0.9 ng/mL as “low normal,” and 0.3 to 0.6 ng/mL as “low” and less than 0.3 ng/mL is considered to be a “very low” range [19]. With age, OR falls to a critical level, finally leading to menopause. AMH has been successfully used in predicting the median age to menopause well beforehand, rendering it as the best endocrine marker to predict the age related decline in OR [20].

## 3. Assessment of Anti-Mullerian Hormone: Above and Beyond

In assisted reproductive technology (ART), optimization of the treatment protocol and counseling of patients require steadfast evaluation of OR. While several studies have used various direct and indirect biochemical measures to provide an insight, none of these fulfill the criteria of a single parameter satisfactory for OR assessment. Inhibin B and AMH are examples of direct measures as they are produced during follicular development, independent of follicular stimulation. Similarly, indirect measures include FSH, LH, and estradiol, as they rely on the production of other hormones through a feedback loop. AFC evaluation on transvaginal ultrasound is a reliable biophysical marker for OR estimation. With the development of more robust and reliable laboratory methods in recent times, AMH is being extensively investigated. Its assessment has several advantages over other biochemical and biophysical markers. Here we have briefly discussed the traditional ovarian markers and have compiled evidence to support the larger role of AMH as opposed to others.

### 3.1. Follicle Stimulating Hormone (FSH)

Traditionally, FSH has been the OR biomarker of choice. It is well studied, documented, and validated and thus provides a level of comfort to physicians. Since the late 1980s, it is being used to indicate hypothalamic-pituitary-gonadal axis functioning. Currently, World Health Organization classifies ovarian dysfunction on the bases of serum FSH and estradiol levels [21]. Testing is available on multiple automated platforms being relatively fast, inexpensive, and reproducible. FSH fluctuates with the menstrual cycle; therefore the samples are collected on day 3 of menstrual cycle to reflect the basal level. As women and their follicles age, FSH rises in reaction to decreased responsiveness of ovary [22].

Even though FSH is the most widely recognized ovarian marker, yet it may not be the best option. For instance, it exhibits both inter- and intracyclic fluctuations; thus single day 3 FSH measurement may fail to be an accurate marker, suggesting evaluation of subsequent cycle's day 3 FSH [23]. Secondly, the assessments of FSH, estradiol, and Inhibin B all suffer from low sensitivity in the initial stages of diminished OR, only becoming abnormal once the reduction in reserve is critically low. Any fertility assistance at this stage of OR may hardly improve the chances of assisted pregnancy [24]. Thirdly, it is affected by several conditions (other than ovarian causes). Therefore, FSH levels are raised in patients receiving hormonal therapy, oral contraceptive pills, and pituitary tumors and in patients with Turner syndrome even in the presence of optimum OR [25, 26]. In contrast, lower levels of FSH are observed in nonovulatory polycystic ovarian syndrome (PCOS) and nonfunctioning pituitary tumors [27, 28]. In all such cases, FSH does not reflect OR consistently; however AMH still reflects the true reserve being independent of hypothalamic axis feedback loop.

These weaknesses can be resolved by assessment of AMH since it stands as the “cycle-independent” marker that remains steady throughout the menstrual cycle [29]. Furthermore, it has emerged as an early indicator of decreased OR as decline in AMH level is reported parallel to drop in the OR. This makes it ideal for screening and timely referring patients to ART clinics [6]. Regarding prediction of menopause, rise in FSH levels, estimation of AFC, and decline in AMH all show significant correlation; however numerous researches have observed greater strength of AMH and AFC as opposed to FSH in this regard [6]. Though FSH has an established role in ART, recent advances highlight the strengths of AMH to fill in gaps encountered with FSH evaluation as an OR marker.

### 3.2. Luteinizing Hormone (LH)

Luteinizing hormone is a glycoprotein, secreted by the anterior pituitary gland. It has a decisive role in ovarian steroidogenesis and ovulation. It progressively increases across the follicular phase of menstrual cycle and peaks at the time of ovulation [30]. LH receptors are more sparsely located on dominant follicle than the smaller follicles, allowing them to continue growing, compared to others [30]. These antral follicles under the influence of LH rapidly synthesized estradiol, essential for oocyte maturation and endometrium readiness for implantation. It is also required for priming of the hypothalamic-pituitary-ovarian axis for successive LH surge required for ovulation induction [31].

This physiological mechanism has prompted its use in assessment of ovarian functions. In 1998, basal day 3 LH concentrations of less than 3 mIU/mL were reported to be the earliest evidence of prediction for a poor ovarian response in ART [32]. Interestingly since then, most of the researchers have found a lack of association between LH concentration and prediction of OR and ART outcomes. In addition, its intra- and intercyclic fluctuation also decrease the power of reproducibility. In patients with PCOS, raised levels of LH are often reported, rendering LH/FSH ratio assessment as a sensitive marker to diagnose PCOS [33]. On the contrary, there is a much stronger evidence of correlation amongst raised AMH and PCOS, as the number of AMH secreting small antral follicles is significantly increased in PCOS [3]. Moreover, specificity and sensitivity of serum AMH in predicting OR in general and the response of ART in particular stand much higher than LH as well as FSH/LH ratio [34]. In a nutshell, in comparison to AMH, LH has a feeble association with ovarian pool and follicular growth as well as response to ovarian stimulation.

### 3.3. Inhibin B

Inhibin B, a heterodimeric glycoprotein, exerts its negative feedback effect on FSH secretion from the pituitary gland, potentiating FSH withdrawal from nondominant follicles [35]. Both AMH and Inhibin B are secreted from the granulosa cells of primary, secondary, and early antral follicles and represent the size of GnRH-reactive antral follicles. To provide a reliable interpretation, Inhibin B is assessed on the same day of cycle (days 2 to 5), as it peaks in early follicular phase and declines to nondetectable levels during luteal phase [36]. Prediction of menopause is critical in older patients who are willing to undergo assisted infertility treatment. Although, Inhibin B levels drastically fall with age, they are less predictive of menopause. On the contrary, researchers suggest that AMH can accurately reflect this transition up to five years prior to finally attaining menopause [37].

Inhibin B has also been studied to predict the outcomes of ART. Conversely, no added advantage has yet been confirmed over FSH as well as AMH, in assessing poor response or ovarian hyperstimulation syndrome (OHSS), number of oocytes retrieved or pregnancy outcomes [38]. Even in PCOS, its levels remain within normal limit, excluding it as a marker of increased follicular growth [38]. It is noteworthy that, in young cancer patients receiving ovarian toxic chemotherapy, Inhibin B and estradiol remain unchanged while AMH falls drastically, with a modest rise in FSH level [39]. Thus chemotherapy induced ovotoxicity is most consistently indicated by assessment of AMH levels.

### 3.4. Estradiol (E2)

Estradiol is a steroid sex hormone, produced by the ovarian follicles as well as adipose, liver, adrenal, breast, and neural tissues [40]. Due to its multiple sites of secretion, it is never evaluated as a solo marker. It is worth mentioning that E2 might be decreased in estrogen-producing tumors and elevated due to precocious puberty, falsely being interpreted for ovarian status [41]. The secretion of E2 by the early antral follicles is under the influence of FSH led hypothalamic dependence, that is, feedback-dependent.

E2 has been used to monitor OR in women with amenorrhea or menstrual dysfunction and to detect the state of hypoestrogenism and menopause. Furthermore, estrogen monitoring is considered useful to assess follicular growth during fertility therapy. There are mixed reports relating elevated basal E2 levels and a poor ovarian response; however few studies have established correlation between poor ovarian response and E2 levels of <20 or >80 pg/mL [42]. Although the levels of circuiting E2 had been successfully used in decreasing the incidence of OHSS, high serum levels of AMH are more strongly associated with OHSS [43].

These findings lead to the conclusion that while comparing the pit falls and clinical applicability of AMH, basal estradiol has a very low predictive accuracy, for both ovarian responsiveness and pregnancy outcomes. AMH being solely secreted by ovaries as well as devoid of the feedback loop fulfills the criterion of “autonomous analyst of OR.”

### 3.5. Antral Follicle Count (AFC)

Antral follicle count is considered as most reliable method to evaluate the ovarian response; however it suffers from operator variability as well as mechanical inconsistency. Even with the same operator, AFC has higher intra- and intercycle variability [44]. It is preferably assessed in early follicular stage, as the presence of larger follicles or corpus luteum interferes in accurate visualization and estimation. As transvaginal ultrasound (TVS) does not differentiate between healthy and atretic follicles, it counts both as capable of responding to treatment [45]. To utilize the real potential of AFC, it is essential to invest in technology as well as training of the staff, in order to obtain accurate clinical interpretation. Furthermore, TVS is not a suitable technique in females with previous ovarian surgeries or ovarian cysts, as they hinder in visualization of small follicles [45]. It is noteworthy that both AFC and AMH appear to be valuable indicators. Studies suggest that AMH relates well with the basal AFC and thus are considered interchangeable [6]. As blood tests clearly have marked advantages over ultrasound for primary care physicians, AMH has a greater efficiency over AFC, especially in setups where high class technology is not available.

In the context of ART, AFC is used in routine to monitor the ovarian responses. Different studies have taken various counts to define normal AFCs (10 ± 4 follicles); thus absence of a standard cutoff or uniform measurement criteria has hampered in accurate clinical interpretation [46]. On the other hand, threshold values of AMH, ranging from 0.2 to 1.26 ng/mL, have been used to identify poor responders with 80–87% sensitivity and 64–93% specificity [47]. AMH may be considered as more sensitive probe to identify early follicles measuring 0.4 to 2 mm, as they are not visualized on TVS. In ovarian stimulation, a combined evaluation of AFCs and AMH has successfully led to prediction of poor as well as hyperresponsiveness [48]. This has opened new horizon to individualized infertility treatment in order to maximize the chance of pregnancy and eliminate iatrogenic side effects for each patient [48].

To summarize, both AFC and AMH have clinical value in providing useful information regarding OR as well as responsiveness to treatment. AMH more accurately reflects very small and nonatretic follicles, reflecting a true picture of OR while AFC helps to visualize the size of growing follicles, crucial to analyze the progress of stimulation. It is clearly evident that analysis of multiple markers has significantly improved predictive accuracy of ART outcomes (Table 1).

## 4. Possible Clinical Applications of AMH

### 4.1. Pediatrics Disorders of Sex Development

AMH has emerged as a marker of gonadal development and reproductive disorders in pediatric age group. As discussed earlier, along with testosterone, it plays an essential role in sex differentiation and the normal development of testes. Its absence or lower levels in males suggest dysfunctional testis while in female, its presence in higher concentration indicates existence of testicular tissue [1]. Currently, AMH is being used to determine the presence of testicular tissue in conditions such as ambiguous genitalia, anorchia, or cryptorchidism [49]. In Klinefelter syndrome, it indicates the severity of testicular dysfunction [50]. In children treated for ovotestis, AMH has the potential of being a diagnostic marker for identifying the presence of testicular tissue, before and after surgical intervention [51]. Moreover, it may assist in differentiating between various causes of virilization in girls. AMH is found to be raised in granulosa cell tumor or testicular tissues induced virilization while it is normal in virilization caused by congenital adrenal hyperplasia [1]. Turner syndrome patients are prone to be at a higher risk of accelerated OR loss; monitoring of AMH in such cases seems to be an excellent indicator of premature ovarian insufficiency, suggesting timely interventions [52].

### 4.2. Obesity Associated Infertility

Obesity affects one-fifth of the female population, with 18.3% belonging to the reproductive age group (16–44 years) [53]. Prevalence of infertility is higher in obese women due to decreased OR as well as follicular dysfunction [54]. Although its underlying mechanism is not well understood, it is hypothesized that low levels of adiponectin (adipocytokine) stimulate aromatase activity in the ovary [55]. As a result, AMH production falls, reflecting dysfunctional folliculogenesis. A number of studies have reported significant relationship between low levels of AMH and higher body mass index (BMI) while few studies reported lack of relationship [53]. This might be due to the fact that the obese patients suffering from underlying pathology such as PCOS have a much higher level of AMH, giving a false overall picture. This fact should be deemed as strength of AMH that truly reflects the status of the ovarian reserve without being influenced by conditions such as obesity.

### 4.3. Assisted Reproductive Technology

Age, FSH, LH, estradiol, Inhibin B, AFC, and ovarian volume have been conventionally used to assess ovarian function for years. These parameters assist in protocol selection and counseling of patients. In the last decade AMH has emerged superior to other markers of OR. As AMH has a significantly better predictive value than FSH and AFC, especially in women over 38 years, AMH evaluation is beginning to establish its place in baseline investigation prior to ovarian stimulation [56]. Infertility clinics are increasingly analyzing patient's age with AMH to design individualized stimulation protocol. In clinical practice, AMH evaluation has guided infertility care physicians to modify the dose of medication, avoiding the risk of OHSS or cycle cancellation due to nonresponsiveness [57]. However, it is important to understand that although AMH is a reliable predictor of ovarian response, yet researches report a lack of predictive accuracy for pregnancy outcomes. This might be due to the fact that it indicates the quantity of the follicle but does not rule out the chance of “compromised quality” [45]. Therefore, patients with higher AMH may still fail to conceive while positive pregnancies have been reported in patients with extremely low AMH [58]. In the recent times, AMH levels within ovarian follicular fluid have been strongly associated with the pregnancy rates in IVF (in vitro fertilization) treatment [2]. In a nutshell, AMH is a useful tool to predict and evaluate the efficacy of the treatment but it has a limited clinical use as a marker of pregnancy outcomes.

### 4.4. Polycystic Ovarian Syndrome (PCOS)

PCOS is the most common cause of anovulatory infertility, affecting 10 to 15% women of reproductive age [59]. In various parts of the world, PCOS is currently being diagnosed on the basis of Rotterdam criteria which diagnoses the syndrome on the presence of at least two of the following three features: hyperandrogenism, oligomenorrhea, and polycystic ovaries [60]. In recent years, it has been reported that, in PCOS, AMH levels are elevated up to two- to threefold, reflecting the load of growing follicles [61]. This correlation with follicular growth implies the strength of AMH as a marker of severity of ovarian dysfunction and hyperandrogenism in women with anovulatory PCOS [61]. In the light of current literature, it seems that AMH will soon secure a place in Rotterdam criteria as a diagnostic marker for PCOS [62].

### 4.5. Chemotherapy and Radiotherapy Induced Ovarian Damage

Groundbreaking advancements in cancer treatment have paved the way to improved survival rates, highlighting the importance to preserve an optimal quality of life after treatment. Ovaries are considered as a major target of xenobiotic which specifically affect the growing follicles. Xenobiotics cause infertility, a worrisome consequence in childhood cancer survivors and women of reproductive age [10]. The pre- and posttreatment analysis of AMH give a useful picture of the damage caused by chemotherapy or pelvic radiation. Similarly, AMH after treatment evaluation also reflects the recovery of gonadal function on completion of the treatment [63]. On the bases of AMH monitoring, timely referral to reproductive endocrinologist can assist childhood cancer survivors in their puberty progression. Donation of ovum is prohibited in various countries; thus AMH is a relevant marker to screen women at a higher risk of developing chemotherapy induced infertility, referring them well in time for ART consultation and fertility preservations.

### 4.6. Ovarian Tumors

In 1992 AMH was identified as the marker of ovarian tumors of granulosa cell origin. As it is exclusively secreted by granulosa cells, it is a reliable marker for diagnosis as well as monitoring for recurrences of tumor. Raised levels have been found in 76 to 93% of women with granulosa cell tumors [2]. Moreover, AMH surge is observed up to 16 months prior to clinical recurrence of the tumor itself, suggesting it as a useful marker of granulosa cell activity [64].

As AMH induces regression of Mullerian duct in fetal life, lately in vitro studies have highlighted its inhibitory role in epithelial cell ovarian cancers [65]. This seems to be a beginning of the newer role of AMH as a therapeutic and/or diagnostic agent (Table 2).

## 5. Conclusion

AMH initially considered as a male hormone has emerged as an invaluable tool for assessment of ovarian function in childhood, adolescence, and adult females. Serum AMH is an autonomous marker reflecting “acyclic ovarian activity.” Strong correlation with follicle numbers, operator independency, and accurate prediction of reproductive lifespan makes it a timely and reliable indicator. In developing countries, AMH evaluation is presently not included in the baseline assessment preceding ovarian stimulation in ART, but in the light of reviewed evidence we propose that inclusion of AMH evaluation can essentially lead to individualization of therapeutic strategy, minimizing iatrogenic effects as well as the liability of the cost. As with conventional OR markers, AMH has low predictive accuracy for live births. Pregnancies have even been reported with very low AMH, indicating its inability to reflect on the quality of oocyte. Hence its use in ART should be aimed for effective designing of protocol and counseling, at the same time keeping in mind that patients should not be deprived of treatment on the ground of very low AMH. Further research on the significance of varying levels of AMH within follicular fluid may pave the way to establish it as a marker of “quality” besides quantity of the growing follicles.

## Figures and Tables

**Table 1:** Comparison of characteristics of the most widely used markers of ovarian reserve (modified with permission from La Marca et al. [44, 47]).

Characteristics of a good marker	Age	AMH	FSH	AFC
Low intercycle variability	+++	+++	−	++
Low intracycle variability	+++	++	−	++
Applicable to all patients	+++	+++	+	+
Operator independency	+++	+++	+++	−
Prediction of poor response	+	+++	++	+++
Prediction of hyper response	+	+++	+	+++
Prediction of oocyte retrieval	++	+++	+	+++
Individualization of treatment	+	+++	−	+++
Economics	+++	−	−	−

−: not appropriate, +: not very appropriate, ++: appropriate, and +++: very appropriate.

**Table 2:** Summary of available evidence for clinical applications of anti-Mullerian hormone.

Year	Author	Sample size	Study	Outcomes
2013	Fleming et al. [66]	*n* = 683 PCOS	Meta-analysis	AMH value of 4.7 ng/mL has the power to diagnose PCOS with (i) 79.4% specificity and 82.8% sensitivity & AUC = 0.87 (95% CI 0.83–0.92)

2011	Karkanaki et al. [2]		Review	Decreased AMH in (i) Ageing, higher BMI, ovariectomy, chemo/radio therapy, GnRH administration, pregnancy, and oral contraceptive pills Raised AMH in polycystic ovarian syndrome

2013	Lindhardt Johansen et al. [1]		Review	Diagnostic role of AMH in pediatric group includes the following: (i) Determination of testicular tissue (ii) Persistent Mullerian duct syndrome (iii) Females with virilization and polycystic ovaries (iv) Premature ovarian insufficiency (v) Hypogonadotropic hypogonadism (vi) Klinefelter syndrome (vii) Granulosa cell tumor

2011	Yates et al. [57]	Case *n* = 423 Control *n* =346	Retrospective	AMH tailor individualized ovarian stimulation significantly (i) Increased embryo transfer (79–87%) and live birth rates (15.9–23.9%) (ii) Decreased OHSS (6.9 to 2.3%) and failed fertilization (7.8 to 4.5%) (iii) Reduced cost of fertility treatment by 29% per patient

2014	Broer et al. [21]		Review	Novel indications for use of AMH for ovarian reserve testing in (i) Small for gestational age (ii) Type I diabetes mellitus (iii) Autoimmune diseases like lupus erythematous (iv) Ovarian surgery and uterine artery embolization for fibroids (v) BRCA 1/2 mutation carriers
